# PHBV/PCL Microparticles for Controlled Release of Resveratrol: Physicochemical Characterization, Antioxidant Potential, and Effect on Hemolysis of Human Erythrocytes

**DOI:** 10.1100/2012/542937

**Published:** 2012-05-01

**Authors:** Jessica Bitencourt Emilio Mendes, Manoela Klüppel Riekes, Viviane Matoso de Oliveira, Milton Domingos Michel, Hellen Karine Stulzer, Najeh Maissar Khalil, Sônia Faria Zawadzki, Rubiana Mara Mainardes, Paulo Vitor Farago

**Affiliations:** ^1^Postgraduate Program in Pharmaceutical Sciences, Department of Pharmaceutical Sciences, State University of Ponta Grossa, 4748 Carlos Cavalcanti Avenue, 84030-900 Ponta Grossa, Brazil; ^2^Laboratory of Quality Control, Department of Pharmaceutical Sciences, Federal University of Santa Catarina, P.O. Box 476, 88040-900 Florianópolis, Brazil; ^3^Laboratory of Nanotechnology, Department of Pharmacy, State University of the Center-West, 3 Simeão Camargo Varela de Sá St, 85040-080 Guarapuava, Brazil; ^4^Department of Materials Engineering, State University of Ponta Grossa, 4748 Carlos Cavalcanti Avenue, 84030-900 Ponta Grossa, Brazil; ^5^Department of Chemistry, Federal University of Paraná, P.O. Box 19081, 81531-990 Curitiba, Brazil

## Abstract

Microparticles of poly(3-hydroxybutyrate-*co*-3-hydroxyvalerate) (PHBV) and poly(**ε**-caprolactone) (PCL) containing resveratrol were successfully prepared by simple emulsion/solvent evaporation. All formulations showed suitable encapsulation efficiency values higher than 80%. PHBV microparticles revealed spherical shape with rough surface and presence of pores. PCL microparticles were spherically shaped with smooth surface. Fourier-transformed infrared spectra demonstrated no chemical bond between resveratrol and polymers. X-ray powder diffraction patterns and differential scanning calorimetry analyses indicated that microencapsulation led to drug amorphization. These PHBV/PCL microparticles delayed the dissolution profile of resveratrol. Release profiles were better fitted to biexponential equation. The hypochlorous-acid-scavenging activity and 2,2-azinobis(3-ethylbenzothiazoline-6-sulfonic acid) radical cation discoloration assay confirmed that the antioxidant activity of PHBV/PCL microparticles was kept, but was dependent on the microparticle morphology and dissolution profile. Resveratrol-loaded PHBV/PCL microparticles showed no cytotoxic effect on red blood cells.

## 1. Introduction

Oxidative damage occurs as an outcome of an imbalance between the formation and inactivation of oxygen free radicals and has a potential to have deleterious effects [1]. Oxidative stress is included as a main cause of several chronic human diseases as cancer, diabetes, hypertension, cardiovascular diseases, and aging [2]. The superoxide anion (O_2_^∙−^), hydrogen peroxide (H_2_O_2_), and hydroxyl radical (OH^∙^) are some of the reactive oxygen species (ROS) and can produce (a) damage to cell membranes or other lipid structures mostly by lipid peroxidation of unsaturated fatty acids, (b) change in proteins by altering the tertiary structure and leading to loss of function, fragmentation, and crosslinking, and (c) changes in DNA which can be rearranged by repair mechanisms or may induce mutations [1, 3].

**Figure 1:**
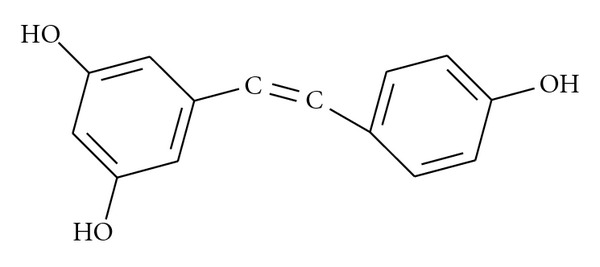
Chemical structure of resveratrol.

 Currently, there is a great interest in antioxidants mainly due to the findings on the remarkable effects of free radicals in the human body. During an oxidative stress, the excess of free radicals can be counteracted by antioxidants produced endogenously or absorbed through the diet [4]. Considering this perspective, resveratrol or *trans*-3,5,4′-trihydroxy-*trans*-stilbene (Figure 1) is a phytoalexin produced by plants in response to exogenous stress factors, such as injury, fungal infections, or UV irradiation. It has been shown to be a potent antioxidant, anti-inflammatory, anticancer, and chemoprotective agent. It is reported that the possible mechanisms for its various pharmacological activities involve modulating lipid metabolism, platelet aggregation, and inflammatory response [5–9]. The properties of resveratrol are attributed to its ability to inhibit low-density lipoprotein oxidation, while suppressing the activity of cyclooxygenase 2 and induced nitric oxide synthase also contributes to the anti-inflammatory and antioxidant effects [10]. This compound can particularly affect the process of carcinogenesis in its three stages: initiation, promotion, and progression. This drug has proved to be a suppressor of angiogenesis and metastasis of tumors [11–14].

 Despite the numerous studies on the *in vitro* properties of polyphenolic compounds, their suitable effects are often not observed *in vivo*. This difference can be partially attributed to a low absorption and a high metabolism of these compounds that lead to a reduced *in vivo* result by oral administration as compared to their great *in vitro* efficacy [15]. Regarding the resveratrol, its polyphenolic structure shows high hydrophobicity and it is sensitive to some external agents such as air, light, and oxidative enzymes that can induce oxidation and a light-induced conversion from the trans (Z) to the cis (E) isomer and can reduce its viability and bioavailability for clinical use [16–18].

Some recent papers are devoted to investigate resveratrol-loaded micro-/nanoparticles in order to provide a controlled release or to improve its stability and bioavailability. Calcium-pectinate beads containing resveratrol were prepared and showed a delayed release and a site-specific delivery to the lower gastrointestinal tract [11]. Experimental parameters were studied to obtain resveratrol-loaded poly(*ε*-caprolactone) nanoparticles with higher encapsulation efficiency and lower particle sizes by oil-in-water emulsion/solvent evaporation method [16]. Inclusion complexes between *trans*-resveratrol and **β**-cyclodextrin or hydroxypropyl-**β**-cyclodextrin revealed improved solubility and kept the scavenging capacity against the stable radical DPPH^∙^ [19]. Resveratrol-loaded lipid-core nanocapsules increased the concentration of *trans*-resveratrol in the brain tissue and can be used for several brain diseases including Alzheimer's disease [20]. Pharmacokinetic studies of resveratrol-loaded nanoparticles were performed and demonstrated an increased systemic bioavailability [21]. Vanillin cross-linked chitosan microparticles showed a controlled release of resveratrol [22].

 Inspite of these papers, the literature does not report studies involving resveratrol-loaded microparticles obtained from poly(3-hydroxybutyrate-*co*-3-hydroxyvalerate) (PHBV) and poly(**ε**-caprolactone) (PCL) as polymers. These polyesters are attractive materials for controlled-release drug applications due to their biodegradability. Their decomposition products are not toxic and are easily excreted [23]. Moreover, a lack of data is available regarding the effect of resveratrol-loaded microparticles on hemolysis of red blood cells. Thus, the aim of this paper was to obtain resveratrol-loaded PHBV/PCL microparticles by simple emulsion/solvent evaporation method and to evaluate their erythrocyte cytotoxicity using the hemolysis assay in order to investigate the feasibility of applying these polyester microparticles as an oral drug delivery carrier for controlled release.

## 2. Materials and Methods

### 2.1. Materials

*trans*-resveratrol (99.8% pure, Pharma Nostra, Rio de Janeiro, Brazil), poly(3-hidroxybutirate-*co*-3-hidroxyvalerate) (PHBV) ($\overset{®}{M_{w}}$ = 380,000 g·mol^−1^, 8.70 mol% hydroxyvalerate, Biocycle L110, PHB Industrial, Serrana, Brazil), poly(**ε**-caprolactone) (PCL) ($\overset{®}{M_{w}}$ = 70,000–90,000 g·mol^−1^, Sigma-Aldrich, St. Louis, MO, USA), and poly(vinyl alcohol) (PVA) ($\overset{®}{M_{w}}$ = 72,000 g·mol^−1^, 88.5 mol% of hydrolysis, Vetec, Rio de Janeiro, Brazil) were used as received. The other reagents and solvents were of analytical grade.

### 2.2. Preparation of Resveratrol-Loaded PHBV/PCL Microparticles

The polyester microparticles containing resveratrol were prepared by simple emulsion/solvent evaporation procedure [23]. Three different formulations (Table 1) were obtained for each polymer (PHBV/PCL) depending on the amount of resveratrol into their compositions (5, 10, and 20%). Chloroform and methylene chloride were used as polymer solvent for PHBV and PCL, respectively. Briefly, the organic phase was added into the aqueous phase under mechanical stirring (5.000 rev·min^−1^) for 5 min. The emulsion was kept under mechanical stirring (800 rev·min^−1^) at room temperature for 4 h. After evaporation of organic solvent, microparticles were separated by centrifugation (2.500 rev·min^−1^, 10 min), washed twice with purified water and dried under vacuum at 35 ± 2°C for 4 h. The samples were stored into a desiccator under vacuum at room temperature. All formulations were obtained in triplicate. Unloaded microparticles were also prepared as negative controls.

### 2.3. Residual Moisture

The water content of resveratrol, PHBV, PCL, and microparticles was performed using an infrared moisture analyzer (Top 160 Ray, Bel engineering, Monza, Italy). For each sample, an amount of 1.000 g was placed on an aluminum plate and dried at 105°C until constant weight. The percentage corresponding to mass loss was obtained as moisture content. The tests were carried out in triplicate.

### 2.4. Characterization

#### 2.4.1. Drug Loading and Encapsulation Efficiency

An amount of microparticles, equivalent to 20 mg of resveratrol, was weighed and magnetic stirred (1,000 rev·min^−1^) with 7 mL ethanol for 12 h. The volume was completed to 10 mL and filtered through a poly(vinylidene fluoride) membrane filter (Durapore membrane, 0.22 *μ*m pore size, Millipore, Bedford, MA, USA). After suitable dilution in ethanol, the concentration of resveratrol was determined through HPLC system (Waters Alliance 2695 HPLC System, Milford, MA, USA) using a Waters XTerra C18 analytical column (250 × 4.6 mm, 5 *μ*m) with UV detection at 306 nm, in triplicate. The mobile phase consisted of 0.2% (v/v) acetic acid in water, acetonitrile, and methanol (2.3 : 22.5 : 75 v/v). The validation of this HPLC method was previously performed through the following parameters: linearity, limit of detection, limit of quantitation, accuracy, robustness, precision, and specificity [24]. The concentration range varied from 10.0 to 50.0 *μ*g·mL^−1^. Linearity was 0.99981, and the detection limit was 172.18 ng·mL^−1^. The encapsulation efficiency (EE) was obtained using(1)

(1) $$\text{EE}=\left(\frac{\text{mass}\;\text{of}\;\text{resveratrol}\;\text{in}\;\text{microparticles}}{\text{theoretical}\;\text{mass}\;\text{of}\;\text{resveratrol}}\right)\times 100.$$

#### 2.4.2. Scanning Electron Microscopy (SEM)

The samples were mounted on aluminum stubs, sputtered with gold (IC-50 Ion Coater, Shimadzu, Kyoto, Japan), and analyzed using a scanning electron microscope (SSX-550 Superscan, Shimadzu, Kyoto, Japan) at an accelerating voltage of 10 or 15 kV with different magnifications.

#### 2.4.3. Particle Size and Size Dispersion

The particle size and size dispersion of PHBV/PCL microparticles were measured by laser diffraction spectrometry (LDS) in a Cilas 920 L apparatus (Marseille, France). The dried powder samples were suspended in filtered water and sonicated into the ultrasonic bath coupled to the equipment for 1 min before measurements. Then, the mean diameters ± standard deviations and the size distributions were determined. The span was calculated using

(2) $$\mathrm{span}=\frac{d_{\left(v,\;90\right)}-d_{\left(v,\;10\right)}}{d_{\left(v,\;50\right)}},$$

where *d*_(*v*,10)_, *d*_(*v*,50)_, and *d*_(*v*,90)_ are the particle diameters determined, respectively, at the 10th, 50th, and 90th percentile of the undersized particle distribution curve.

#### 2.4.4. Fourier-Transformed Infrared Spectroscopy

The Fourier-transformed infrared (FTIR) spectra of raw materials, PHBV/PCL microparticles, and physical mixtures were recorded from 4000 to 400 cm^−1^ on a Shimadzu IR Prestige-21 spectrophotometer (Kyoto, Japan) using KBr pellets with 32 scans and resolution of 4 cm^−1^.

#### 2.4.5. X-Ray Powder Diffraction

Wide-angle X-ray powder diffraction (XRPD) was performed with a Shimadzu X-ray diffractometer (Shimadzu XRD-6000, Kyoto, Japan). The 2*θ* was increased from 5° to 80° at a scan rate of 2°·min^−1^ using a Cu-K*α* source (*λ* = 1.5418 Å) at 40 kV and 40 mA.

#### 2.4.6. Thermal Analyses

**Thermogravimetric Analysis (TG)**

The thermogravimetric curves were obtained in a thermobalance (TGA-50, Shimadzu, Kyoto, Japan) in the temperature range of 298–1173 K using platinum crucibles with 5.0 ± 0.1 mg of sample under dynamic N_2_ atmosphere (flow rate: 50 mL·min^−1^) and heat flow of 10 K·min^−1^. The equipment was previously calibrated with copper sulphate pentahydrate.

**Differential Scanning Calorimetry (DSC)**

DSC curves of resveratrol, PHBV, PCL, physical mixtures, and microparticles were obtained in a DSC-60 calorimeter (Shimadzu, Kyoto, Japan) using aluminum crucibles with 2.5 ± 0.1 mg of sample under dynamic N_2_ atmosphere (flow rate: 50 mL·min^−1^). The temperature range was 298–773 K with heating rate of 10 K·min^−1^. An empty aluminum pan was used as reference. The DSC cell was calibrated with indium (m.p. = 429.6 K; Δ*H*_fusion_ = 28.54 J · g^−1^) and zinc (m.p. = 692.6 K).

### 2.5. *In Vitro* Drug Release

*In-vitro *release was carried out for pure drug and resveratrol-loaded PHBV/PCL microparticles. Dissolution assays were performed in a Nova Ética dissolution tester (299/6, Vargem Grande Paulista, Brazil) equipped with paddles (apparatus II) in 900 mL of degassed phosphate buffer solution (pH = 6.8, 50 mmol·L^−1^ KH_2_PO_4_ and 22.4 mol·L^−1^ NaOH) for 12 h in triplicate. System was kept at a thermostatically controlled temperature of 37 ± 0.5°C and stirred at 75 rev·min^−1^. All experiments were held under dark conditions. At predetermined time intervals, samples were collected (10 mL), filtered (0.45 *μ*m pore size), and spectrophotometrically evaluated (Genesys 10S spectrophotometer, Thermo Scientific, Madison, WI, USA) at 306 nm. The dissolution value was obtained from the amount of drug released. A correction factor was applied to the cumulative dilution caused by replacement of the sample with an equal volume of fresh medium.

#### 2.5.1. Analysis of Release Behavior

Dissolution profiles of resveratrol and PHBV/PCL microparticles were compared by independent and dependent methods as summarized in Table 2. As model-independent analysis, dissolution efficiency, the area under a dissolution curve between defined time points [25], was used. Profiles were also investigated by model-dependent methods using the MicroMath Scientist 2.01 software (Salt Lake City, UT, USA). Data were tested to fit first-order, biexponential, zero-order, and Weibull's equations (Table 2) [26]. The selection of model-dependent method was based on the best correlation coefficient (*r*), the best model selection criteria (MSC), and the best graphic adjustment.

 In order to have some insight into the drug release mechanism, a very simple and semiempirical equation to describe drug release from polymeric systems, the power law (Korsmeyer-Peppas model) [27], was also applied

(3) $$ft=at^{n}$$

where *ft* is the drug dissolved fraction at time *t*, *n* is the release exponent, indicative of the mechanism of the drug release, and *a* is the constant incorporating structural and geometric characteristics of the drug dosage form.

### 2.6. Antioxidant Potential

In order to compare the antioxidant capacity of pure resveratrol and resveratrol-loaded microparticles, the antioxidant potential was evaluated by hypochlorous-acid- (HOCl-) scavenging assay and 2,2-azinobis(3-ethylbenzothiazoline-6-sulfonic acid) (ABTS) radical cation discoloration. Aqueous solutions of pure drug and loaded microparticles (M1R20/M2R20) containing the same concentrations of resveratrol were prepared 30 min before start of experiments and kept under dark conditions.

#### 2.6.1. HOCl Scavenging Activity

After 5 min of reacting 75 *μ*mol·L^−1^ HOCl (30 *μ*L) with each aqueous solution (20 *μ*L) of pure drug or loaded microparticles (M1R20/M2R20), a volume of 20 *μ*L of a solution containing 10 mmol·L^−1^ 3,3′,5,5′-tetramethylbenzidine (TMB) dissolved in 50% dimethylformamide, 100 *μ*mol·L^−1^ potassium iodide and 400 mmol·L^−1^ acetic acid was added [28]. The reaction was performed in 50 mmol·L^−1^ sodium phosphate buffer solution (pH = 7.4) to a final volume of 200 *μ*L at 37°C for 15 min. The absorbance was recorded at 650 nm in a microplate reader (SpectraMax 190 spectrophotometer, Molecular Devices, Sunnyvale, CA, USA). All measurements were performed in triplicate, and unloaded-microparticles (M1R0/M2R0) were used as negative control. The antioxidant activity was obtained as percentage of HOCl inhibition using

(4) $$\text{\%}\;\text{inhibition}=\;\left[\frac{\left(Ab-Aa\right)}{Ab}\right]\times 100$$

where *Ab* is the absorbance of the control and *Aa* is the absorbance of the sample.

#### 2.6.2. ABTS Radical Cation Discoloration Assay

In brief, aqueous solutions of 7 mmol·L^−1^ ABTS and 2.45 mmol·L^−1^ potassium persulfate were mixed in a volume ratio of 1 : 1 and incubated in dark at room temperature for 12 h to obtain ABTS^∙+^ [29]. The ABTS^∙+^ solution was diluted to an absorbance value of ±0.7 at 734 nm in 50 mmol·L^−1^ sodium phosphate buffer solution (pH = 7.4). The reduction between ABTS^∙+^ and pure drug or loaded microparticles (M1R20/M2R20) was measured by decreasing absorbance at 734 nm after 30 min in a microplate reader (SpectraMax 190 spectrophotometer, Molecular Devices, Sunnyvale, CA, USA). All measurements were performed in triplicate, and, unloaded microparticles (M1R0/M2R0) were used as negative control. The antioxidant activity was calculated as percentage of ABTS^∙+^ inhibition according to (4).

### 2.7. Erythrocyte Hemolysis Assay

The *in vitro* cytotoxic effect of pure resveratrol and PHBV/PCL microparticles was studied using heparinized venous blood samples collected from healthy volunteers. This experiment involving human blood was approved by the Ethics Committee of the State University of Ponta Grossa.

 Fresh blood was centrifuged (4000 rev·min^−1^ for 5 min) at 4°C using a refrigerated centrifuge, and the plasma and buffy coat were carefully removed by aspiration. The red blood cells were washed three times by centrifugation (4000 rev·min^−1^ for 5 min) in cold phosphate buffer solution (0.15 mmol·L^−1^ NaCl, 50 mmol·L^−1^ NaH_2_PO_4_/Na_2_HPO_4_, pH = 7.4). The red blood cells were finally resuspended with the same buffer to obtain a hematocrit of 5% [30].

 Freshly prepared aqueous solutions (10 *μ*L) of pure drug or loaded microparticles (M1R20/M2R20) containing the same concentration of resveratrol (71 *μ*g·mL^−1^) were incubated in triplicate at 37°C with a 5% red blood cell suspension (450 *μ*L) and the previously prepared phosphate buffer solution (40 *μ*L) for 24 h under constant shaking at 120 rev·min^−1^. The red blood cell suspension was then centrifuged at 4000 rev·min^−1^ for 5 min. Hemolysis was determined by measuring the absorbance at 540 nm in a microplate reader (SpectraMax 190 spectrophotometer, Molecular Devices, Sunnyvale, CA, USA) [31, 32]. Tests were also carried out for unloaded-microparticles (M1R0/M2R0). Hemolysis observed in the absence of sample was taken as blank. Groups were compared using one-way ANOVA followed by Tukey's *post hoc* test. A *P* value of ≤0.05 was used to indicate statistically significant differences.

## 3. Results and Discussion

The polyester microparticles were successfully prepared by simple emulsion/solvent evaporation method. After drying, PHBV microparticles (system M1) showed powder aspect and pale yellow color similar to PHBV. For PCL microparticles (system M2), powder aspect and off-white color were observed. Water contents of 1.20 ± 0.12%, 3.59 ± 0.17%, and 1.12 ± 0.09% were obtained for pure resveratrol, PHBV and PCL, respectively. Microparticles showed only residual moisture values as presented in Table 3. These data demonstrate that the performed vacuum drying was able to remove the water used during microencapsulation. The residual water content in a powder influences its physical stability and controls the magnitude of capillary forces that hold particles in aggregates [33]. Considering the obtained results, it is possible to establish that the water content exhibits little interference in the physical stability of PHBV/PCL microparticles.

### 3.1. Drug Loading and Encapsulation Efficiency

The drug content and encapsulation efficiency for the prepared microparticles are also summarized in Table 3. High percentages of drug entrapment were obtained for PHBV/PCL microparticles by simple emulsion/solvent evaporation. All formulations showed suitable EE values higher than 80%. These values are mainly based on the poor aqueous solubility (13.6 *μ*g·g^−1^ in pH = 7.4) of resveratrol [34] which leads to an increase in the drug loaded into microparticles. Similar results were previously reported. A resveratrol entrapment higher than 97.7% was achieved for calcium-pectinate beads prepared by instantaneous gelation of pectin [11]. Resveratrol-loaded nanoparticles showed EE from 78.3 to 91.4% using PCL of $\overset{®}{M_{w}}$ = 65,000 g·mol^−1^ [16]. Vanillin cross-linked chitosan microparticles containing resveratrol revealed a drug entrapment higher than 93.7% [22].

 The polymer : drug ratio is also a critical factor during microparticle formation and can influence EE values [35]. The enhancement of resveratrol entrapment was observed when the polyester amount was increased (Table 3). For PHBV microparticles, EE was increased from 80 to 93% as polymer:drug ratio was improved from 4 : 1 (20% resveratrol) to 19 : 1 (5% resveratrol). Resveratrol entrapment varied from 88 to 101% for PCL microparticles when polymer : drug ratio was changed at the same proportion. This effect can be simply due to the greater polymer with respect to the drug amount.

### 3.2. Scanning Electron Microscopy

**Figure 2:**
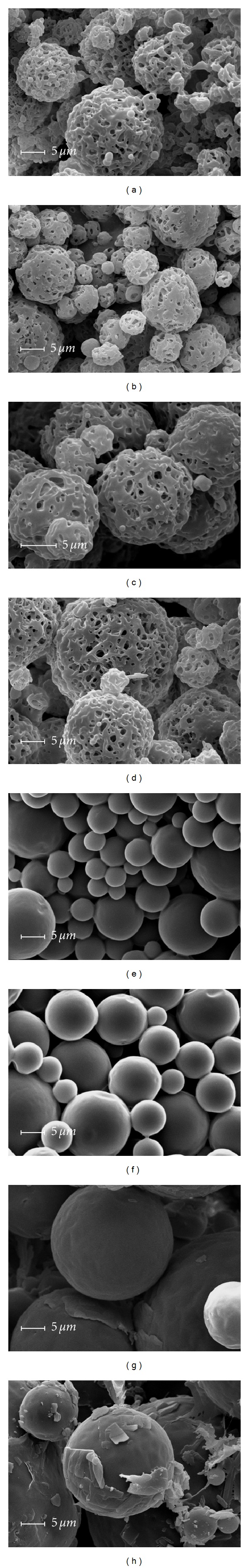
Scanning electron micrographs of PHBV/PCL microparticles: M1R0 (a), M1R5 (b), M1R10 (c), M1R20 (d), M2R0 (e), M2R5 (f), M2R10 (g), and M2R20 (h). Magnifications of 2000x.

The scanning electron micrographs of PHBV/PCL microparticles are shown in Figure 2. Different morphological aspects were observed depending on the polyester used. By SEM, PHBV microparticles were spherical shaped with a rough surface and pores (Figures 2(a)–2(d)). The presence of pores represents important morphological evidence that can change the drug release process from microparticles [36]. However, PCL microparticles revealed a spherical shape with smooth surface (Figures 2(e)–2(h)), and no pore were observed. Moreover, formulation M2R20 showed a residual resveratrol onto the microparticles surface. This external drug can be rapidly dissolved into an aqueous medium and provide an immediate-release behavior (burst effect) [23]. This effect has been also observed in other studies related to delayed-release biopolymer systems [37, 38].

### 3.3. Particle Size and Size Dispersion

The particle size and size distribution obtained by LDS measurements are indicated in Table 3. Micrometer-sized particles with mean diameters less than 60 *μ*m were obtained. Although these particle sizes do not allow an uptake by intestinal tissue, the oral administration of these microparticles can provide sustained drug effect due to their prolonged bowel transit time [39]. Regarding that a low span value indicates a narrow polydispersity [40], PCL microparticles presented a more homogeneous size distribution when compared to PHBV microparticles.

### 3.4. Fourier-Transformed Infrared Spectroscopy

**Figure 3:**
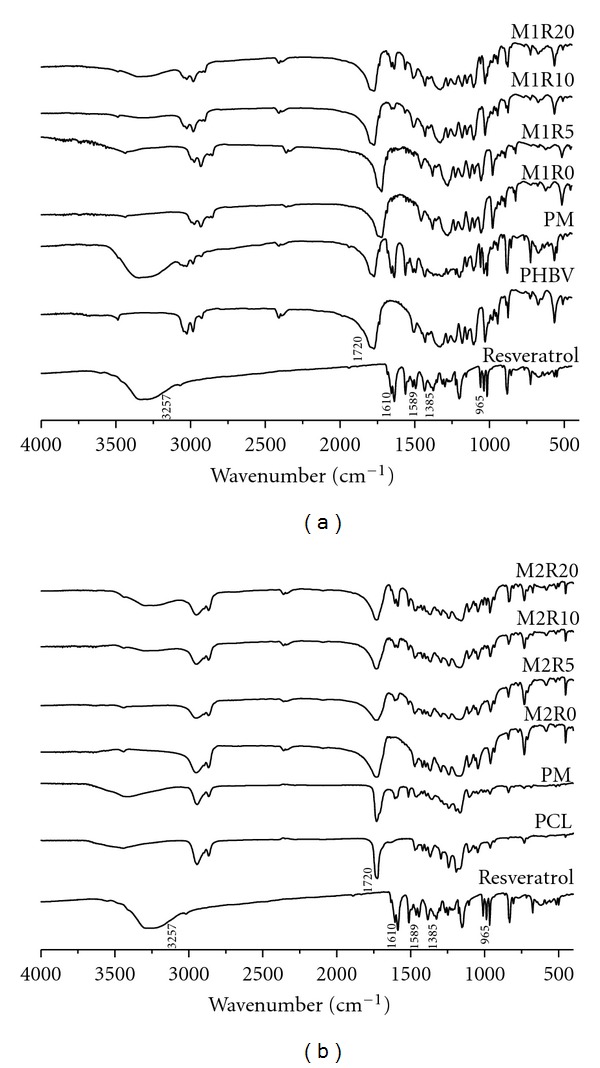
FTIR spectra of resveratrol, PHBV/PCL, physical mixtures, and PHBV/PCL microparticles.

FTIR spectra performed for resveratrol, PHBV, PCL, physical mixtures, and PHBV/PCL microparticles are shown in Figure 3. The FTIR spectrum of pure resveratrol showed a typical OH-stretching band at 3257 cm^−1^ and three intense bands at 1610, 1589, and 1385 cm^−1^ corresponding to C–C aromatic double-bond stretching, C–C olefinic stretching, and C–O stretching. The typical trans olefinic band was observed at 965 cm^−1^ [5]. PHBV infrared spectrum exhibited a strong band at 1720 cm^−1^ due to C=O stretching. Typical bands from 800 to 975 cm^−1^ corresponded to symmetric–C–O–C–stretching vibration. Moreover, the antisymmetric–C–O–C–stretching leads to bands between 1060 and 1150 cm^−1^ [35]. Considering PCL is also an aliphatic polyester, its spectrum is similar to that of PHBV with a strong band at 1727 cm^−1^ corresponding to C=O stretching and two bands at 2943 and 2864 cm^−1^ due to symmetric and asymmetric CH_2_-stretching, respectively [41].

 Physical mixtures and microparticles presented band assignments at the same wavenumber range of FTIR spectrum. Therefore, no difference in the positions of the absorption bands was observed between resveratrol-loaded microparticles and respective physical mixtures. Consequently, no chemical bond between drug and polymers was formed during microencapsulation.

### 3.5. X-Ray Powder Diffraction

**Figure 4:**
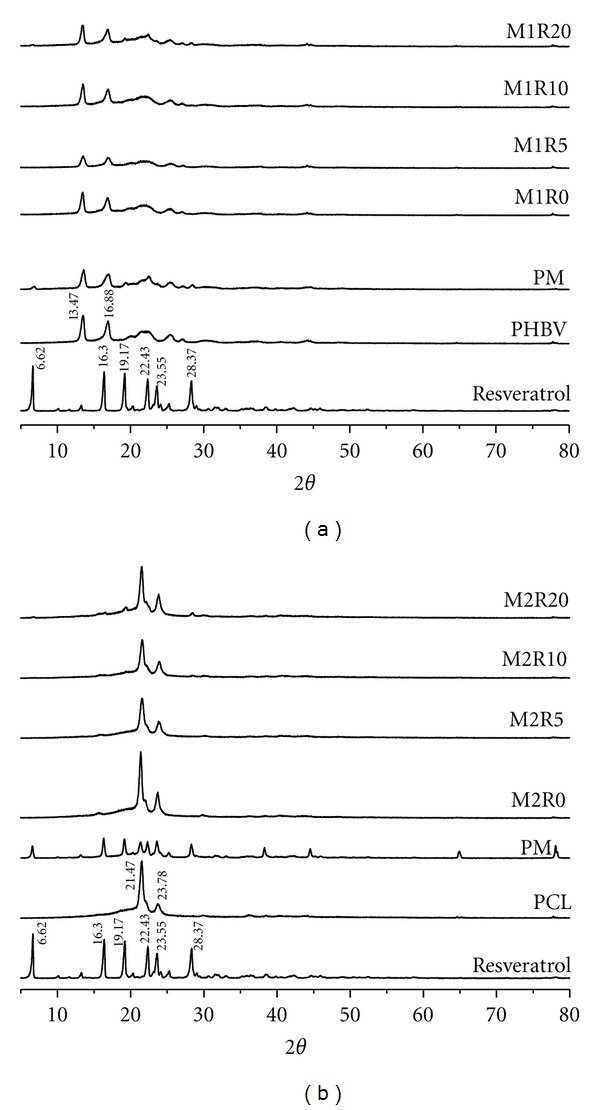
XRPD patterns of resveratrol, PHBV/PCL, physical mixtures, and PHBV/PCL microparticles.

Figure 4 shows the XRPD patterns of resveratrol, PHBV, PCL, physical mixtures, and PHBV/PCL microparticles. Pure resveratrol presented different peaks related to a crystalline structure, and its main peaks appear at 2*θ* = 6.62, 16.30, 19.17, 22.43, 23.55, and 28.37. Resveratrol-loaded microparticles revealed XRPD patterns similar to pure PHBV/PCL and unloaded-microparticles (M1R0/M2R0). These results suggest that the microencapsulation procedure provided a remarkable decrease of the crystalline diffraction peaks of resveratrol leading to drug amorphization [42].

 Substances in solid state can reveal crystalline and/or amorphous characteristics. In general, amorphous solids are more soluble than crystalline forms due to free energies involved in the dissolution process. Solids in amorphous state have randomly arranged molecules, and thus low energy is required to separate them. Consequently their dissolution is faster than when in the crystal form [43]. 

### 3.6. Thermal Analyses

#### 3.6.1. Thermogravimetric Analysis

**Figure 5:**
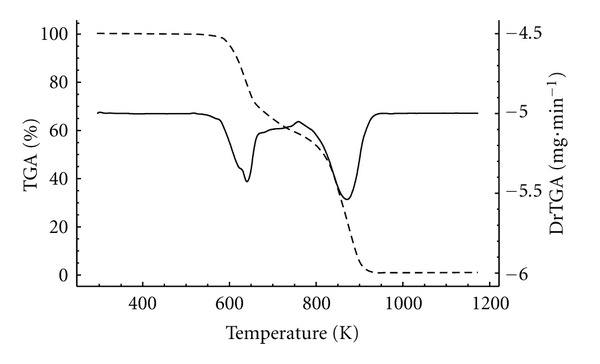
TG and DTG curves of resveratrol.

**Figure 6:**
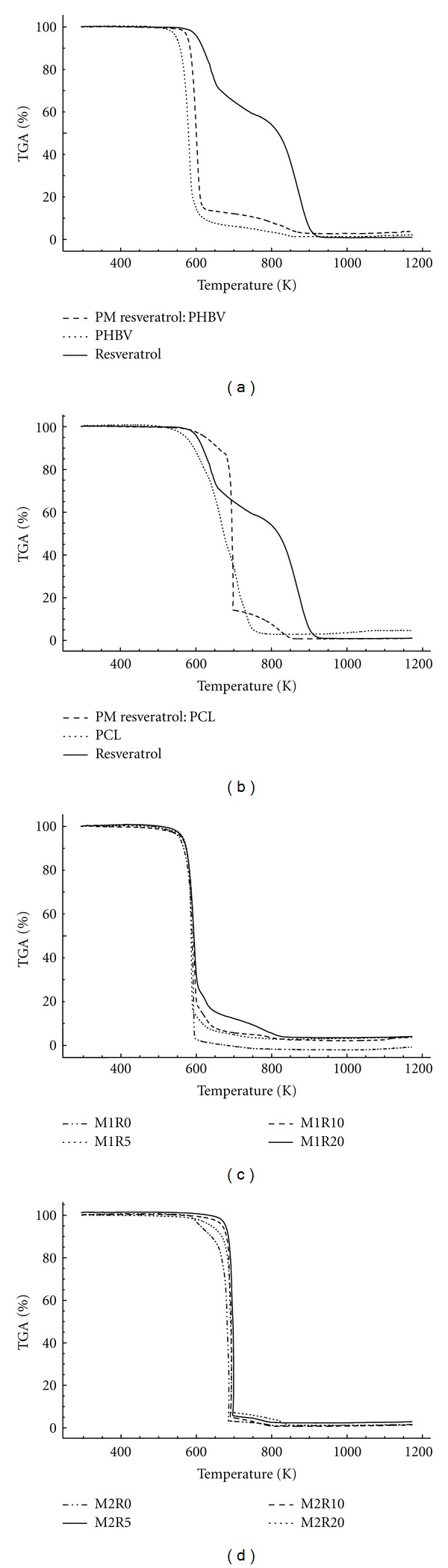
TG curves of resveratrol, PHBV/PCL and physical mixtures (a) and (b), PHBV microparticles (c), and PCL microparticles (d).

The TG curves of pure resveratrol, PHBV, PCL, physical mixtures, and PHBV/PCL microparticles are shown in Figures 5 and 6. Resveratrol presented two events of weight loss that can be observed through its derivative thermogravimetric curve (DTG). The first event ranged from 625 to 758 K (Δ*m* = 41.7%), while the second one occurred between 758 and 1150 K (Δ*m* = 57.5%). Both polymers showed only one event of weight loss, ranging from 564 to 591 K (Δ*m* = 97.6%) for PHBV and from 639 to 734 K (Δ*m* = 97.7%) for PCL.

PHBV microparticles demonstrated onset temperatures of degradation at 585, 581, 577, and 582 K for M1R0, M1R5, M1R10, and M1R20, respectively. Otherwise PCL microparticles started their degradation at 671 K (M2R0), 689 K (M2R5), 686 K (M2R10), and 683 K (M2R20). These results indicate that PCL microparticles are thermally more stable than those composed by PHBV. Similar data were previously described for carvedilol-loaded microparticles, in which formulations containing PCL revealed increased thermal stability than formulations prepared with PHBV [23].

#### 3.6.2. Differential Scanning Calorimetry

**Figure 7:**
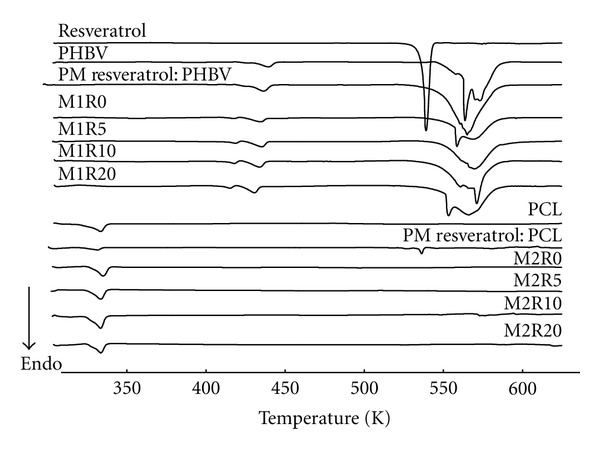
DSC curves of resveratrol, PHBV/PCL, physical mixtures, and PHBV/PCL microparticles.

DSC curves of pure resveratrol, PHBV, PCL, physical mixtures, and PHBV/PCL microparticles are indicated in Figure 7. Resveratrol showed a sharp endothermic event at 539 K in accordance with the literature values [44, 45]. The melting temperatures (T_m_) obtained for polymers were 438 K for PHBV and 333 K for PCL, confirming previously reported data [41]. The typical melting event of resveratrol was not observed in DSC curves of PHBV/PCL microparticles. This thermal behavior suggests that a drug amorphization occurred. This result is reinforced by the XRPD patterns of PHBV/PCL microparticles in which only the characteristic crystalline peaks of the polymers were observed.

### 3.7. *In Vitro* Drug Release and Analysis of Release Behavior

**Figure 8:**
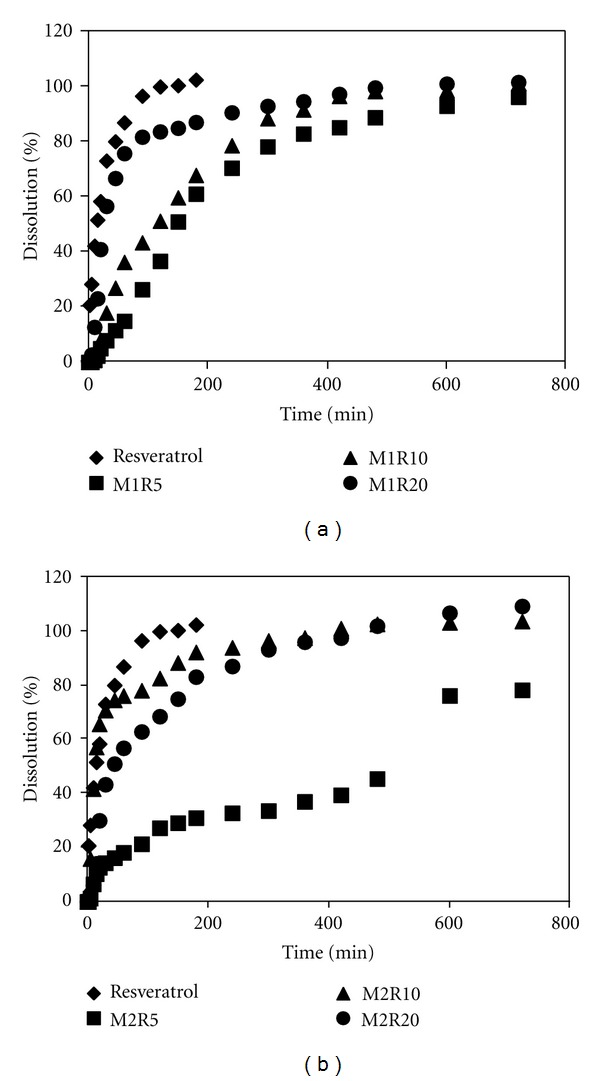
*In vitro* release profiles of pure drug and resveratrol-loaded PHBV/PCL microparticles.

The dissolution rates of resveratrol and PHBV/PCL microparticles are shown in Figure 8. By the performed dissolution test, the mean time for 80% release of pure resveratrol was 45 min. However, PHBV microparticles demonstrated mean dissolution times of 300 min (M1R5), 240 min (M1R10), and 90 min (M1R20) for 80% drug release. For PCL microparticles, a value of 80% drug release was achieved in mean dissolution times of 720, 300, and 180 min for M2R5, M2R10, and M2R20, respectively. Therefore resveratrol from PHBV/PCL microparticles showed a slower dissolution rate than pure drug.

These results demonstrate that PHBV/PCL played an important role on the delay of drug dissolution. This behavior can be related to polymer : drug ratio and morphological aspects. For both PHBV/PCL microparticles, formulations obtained at a polymer : drug ratio of 19 : 1 (5% resveratrol) showed slower release of resveratrol. Probably the greater amount of polyester in these formulations had a remarkable effect in controlling the drug release rate. This faster release of resveratrol from PHBV microparticles can also be related to their porous surface previously observed by SEM. In general, microparticle studies have showed that the drug release rate is faster for higher-porosity materials [41].

 Regarding the dissolution efficiency (DE), microparticles decreased this value extensively. Whereas the pure drug presented a DE of 96.2% along 720 minutes, PHBV microparticles showed DE of 73.3% (M1R5), 78.6% (M1R10), and 88.9% (M1R20). For PCL microparticles, DE of 52.2, 78.0, and 85.0% was obtained for M2R5, M2R10, and M2R20, respectively. Previous papers reported that a high DE was verified for pharmaceutical dosage forms of immediate release while a lower value was indicative of a controlled release behavior [46, 47].

 The release profiles were fitted to mathematical models, and the selection of the best model considered the correlation coefficient (*r*), the model selection criteria (MSC), and the graphic adjustment. Resveratrol and PHBV/PCL microparticles were better fitted to the biexponential equation (Table 4) than other models. The burst-release apparent rate constant (*α*) and the slow-release apparent rate constant (*β*) for resveratrol and PHBV/PCL microparticles are reported in Table 4.

 These results demonstrated that PHBV/PCL microparticles reduced the drug dissolution rate, nevertheless without changing its release model. The first stage of release was initially rapid (burst release) whereas the second stage of release was slow (controlled release). The burst release can help to reach the effective concentration of resveratrol rapidly in plasma, whereas the controlled release would maintain the effective concentration of drug in plasma for a long time [22]. Moreover the poor bioavailability of resveratrol due to its rapid metabolism, including its conjugation with sulfate in the intestinal mucosa, and elimination could be partially avoided by microencapsulation, thus prolonging its biological half-time *in vivo*.

 Concerning the mathematical modeling fitting the Korsmeyer-Peppas model, PHBV microparticles showed *n* values of 1.23 (M1R5), 0.89 (M1R10), and 1.28 (M1R20). For PCL microparticles, *n* values of 0.65, 0.57, and 0.73 were obtained for M2R5, M2R10, and M2R20, respectively. PHBV microparticles presented values of *n* greater than 0.85 indicating that the release mechanism is governed by erosion [27]. Considering that PHBV is an aqueous-insoluble polymer, the process can occur by sequential stages of entrance of water and drug release. Otherwise PCL microparticles revealed values of *n* between 0.43 and 0.85. These intermediate values are related to an anomalous behavior, a non-Fickian kinetics corresponding to the superposition of diffusion and erosion phenomena [27].

### 3.8. Antioxidant Potential

In order to explore whether the microencapsulation has influence on antioxidant capacity of resveratrol, HOCl-scavenging activity and ABTS radical cation discoloration assay of pure resveratrol and resveratrol-loaded microparticles at the same drug concentrations were compared.

**Figure 9:**
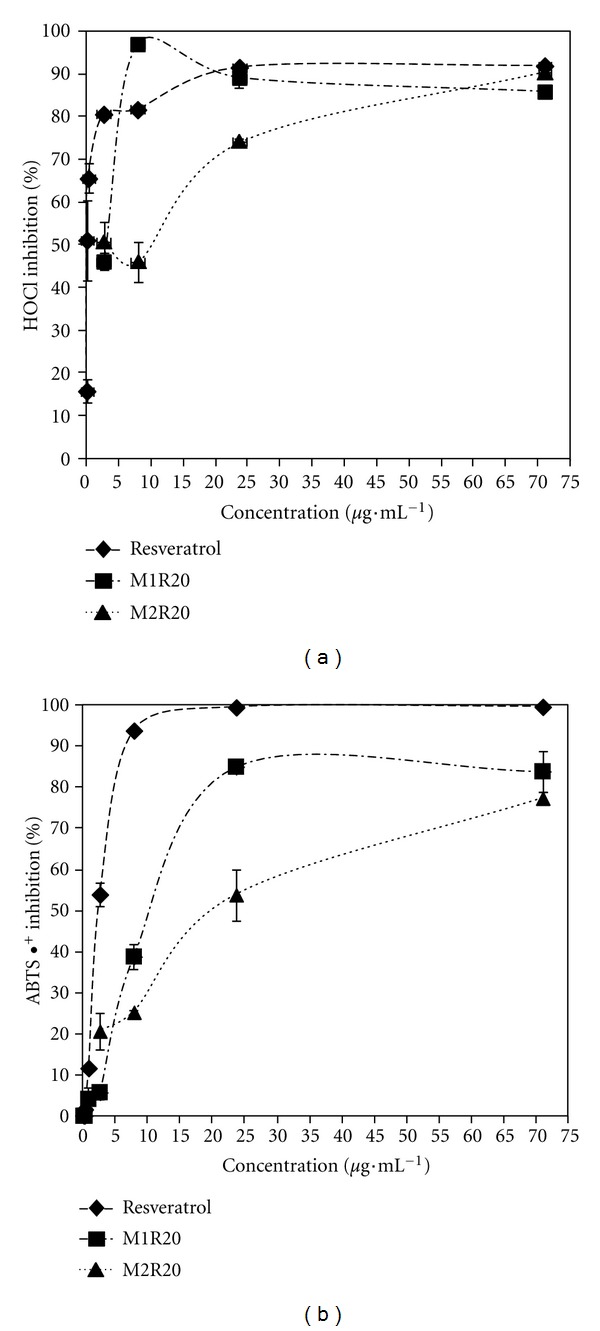
Antioxidant effect of pure resveratrol, and PHBV/PCL microparticles by HOCl- (a) and ABTS^∙+^- (b) scavenging activity.

 The HOCl-scavenging potential of pure resveratrol and PHBV/PCL microparticles is presented in Figure 9(a). Resveratrol (IC_50_ = 0.08 *μ*g·mL^−1^) and M1R20 (IC_50_ = 3.03 *μ*g·mL^−1^) showed higher effect on HOCl scavenging activity, and were followed by M2R20 (IC_50_ = 9.84 *μ*g·mL^−1^). In biological systems, HOCl is the most toxic and abundant oxidizing agent produced by polymorphonuclear neutrophils. It can also attack important biological molecules and generate other harmful ROS.

 The results for ABTS radical cation discoloration test are shown in Figure 9(b). Considering ABTS^∙+^- scavenging potential, a higher activity was obtained for resveratrol (IC_50_ = 2.79 *μ*g·mL^−1^) and M1R20 (IC_50_ = 11.85 *μ*g·mL^−1^). An IC_50_ value of 21.50 *μ*g·mL^−1^ was observed for formulation M2R20. This assay is widely used for screening the antioxidant properties of different compounds and reflects their capacity to donate electrons or hydrogen for inactivating this radical. In both assays, the unloaded microparticles presented a negligible antioxidant effect.

 Regarding the studied concentrations, pure drug and resveratrol-loaded PHBV/PCL microparticles showed different efficiencies in scavenging capacity. Although the results indicated a lower antioxidant activity for resveratrol from PHBV/PCL microparticles, it is important to remember that scavenging assays were performed about 1 h after preparation of aqueous solutions under test, and resveratrol entrapped into polyester microparticles was not completely available to react with oxidizing agents. Therefore PHBV/PCL microparticles have an antioxidant effect even more promising for pharmaceutical purposes than pure drug because of their potential as a controlled-release carrier for prolonging *in vivo* bioavailability of resveratrol. In Despite its high antioxidant activity, pure resveratrol can present a less appropriate behavior biologically due to its rapid metabolism and elimination.

 Morphology of PHBV/PCL microparticles and drug dissolution rate are also strongly related to these antioxidant activities. PHBV microparticles had porous surface which can provide a faster release of resveratrol as compared to PCL microparticles. This porous aspect can also permit a better access of oxidizing species to the resveratrol entrapped into PBHV microparticles. These antioxidant values are in accordance to the *in vitro* drug release profiles, since PCL microparticles revealed the slower dissolution rate.

 Different data were previously reported for inclusion complexes between *trans*-resveratrol and **β**-cyclodextrin or hydroxypropyl-**β**-cyclodextrin that showed minor differences on scavenging capacity against an artificial oxidant, DPPH^∙^ [19]. Otherwise the scavenging activity of resveratrol was reduced when lipid emulsion and aqueous micelle system were prepared [34].

 Thus, it is possible to suggest that the resveratrol-loaded PHBV/PCL microparticles offer a feasible system to control resveratrol release as result of keeping its antioxidant effect.

### 3.9. Erythrocyte Hemolysis Assay

**Figure 10:**
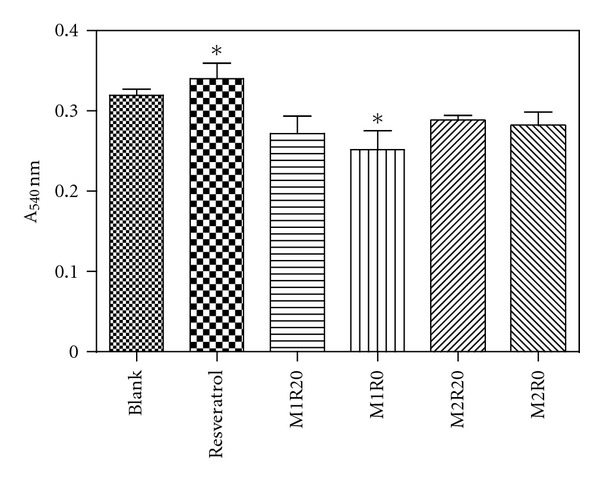
Absorbance values resulting from the released hemoglobin in erythrocyte hemolysis assay for blank, pure resveratrol, and PHBV/PCL microparticles. The asterisk indicates statistically significant difference (*P* = 0.0467).

Hemolysis assay is a fast, efficient, simple, and low-cost procedure to investigate the cytotoxicity of micro-/nanoparticles on erythrocytes membrane by spectrophotometric measurement of the released hemoglobin [48]. The obtained results were presented in Figure 10. Regarding the hemolysis values, pure resveratrol and PHBV/PCL microparticles showed no significant differences to blank (*P* > 0.05). These data support that pure drug and PHBV/PCL microparticles particularly have compatibility with erythrocytes. A statistically significant difference was only observed between resveratrol and formulation M1R0 (*P* = 0.0467).

According to the literature, resveratrol exhibited no hemolytic effect to human red blood cells up to 100 *μ*g·mL^−1^ of concentration [49]. However no previous paper performed the erythrocyte hemolysis assay of resveratrol-loaded and resveratrol-unloaded PHBV/PCL microparticles. Therefore this cytotoxicity assay results demonstrate that the obtained polyester microparticles have no hemolytic properties on human red blood cells which indicate their potential for pharmaceutical and biotechnological applications.

## 4. Conclusion

Polyester microparticles containing resveratrol were successfully prepared by simple emulsion/solvent evaporation. High drug-loading efficiencies were obtained for PHBV/PCL microparticles. Morphological data played a crucial role in evaluating the drug release. Fourier-transformed infrared spectra indicated no chemical bond between resveratrol and polymers. X-ray powder diffraction patterns and differential scanning calorimetry analyses demonstrated that microencapsulation led to a drug amorphization. PHBV/PCL microparticles provided a substantial decrease of dissolution rate of resveratrol without changing its biexponential release model. The antioxidant potential of resveratrol entrapped into PHBV/PCL microparticles was kept, but was dependent on the microparticle morphology and dissolution profile. PHBV/PCL microparticles showed no cytotoxic effect on red blood cells. These results support an experimental basis for the use of resveratrol-loaded PHBV/PCL microparticles as a feasible oral drug delivery carrier for controlled release of resveratrol, being an attractive alternative in chronic diseases prevention.

## Figures and Tables

**Table 1:** Composition of resveratrol-loaded PHBV/PCL microparticles.

Composition	Formulation			
	R0	R5	R10	R20
*Aqueous phase*				
Polysorbate 80 (g)	0.25	0.25	0.25	0.25
PVA (g)	4.00	4.00	4.00	4.00
Purified water (mL)	200.0	200.0	200.0	200.0
*Organic phase*				
Resveratrol (g)	—	0.10	0.20	0.40
PHBV (system M1) or PCL (system M2) (g)	2.00	1.90	1.80	1.60
Chloroform or methylene chloride (mL)	40.0	40.0	40.0	40.0

**Table 2:** Mathematical models related to dissolution experiments.

Model	Equation
Dissolution efficiency	DE = (∫_0_^*t*^ *y* · *dt*/*y*_100_ · *t*) × 100%
First-order	%*D* = 100(1 − *e* ^−*kt*^)
Biexponential	%*D* = 100[1 − (*Ae* ^−*αt*^ + *Be* ^−*βt*^)]
Zero-order	%*D* = *kt*
Weibull	%*D* = 100[1 − *e* ^−(*t*/*TD*)*b*^]

Legend: %*D*: dissolved percentage, *b*: shape parameter, *TD*: time interval necessary to release 63.2% of the drug, *k*, **α** and **β**: kinetics constants, *t*: dissolution time, *A* and *B*: initial drug concentrations that contribute for the two dissolution stages.

**Table 3:** Water content^1^, resveratrol entrapped into microparticles^1^, encapsulation efficiency (EE)^2^, particle size, and span for PHBV/PCL microparticles.

Microparticles	Water content (%)	Resveratrol-loaded (mg·g^−1^)	EE (%)	Mean diameter (*μ*m)	Span
M1R0	1.29 ± 0.11	—	—	25.23	2.38
M1R5	1.50 ± 0.09	46.43 ± 1.77	93	47.00	1.88
M1R10	1.10 ± 0.08	83.80 ± 2.41	84	33.75	2.25
M1R20	1.40 ± 0.12	159.30 ± 2.72	80	29.20	2.27
M2R0	1.57 ± 0.13	—	—	33.97	1.33
M2R5	1.56 ± 0.11	50.63 ± 1.61	101	52.09	1.27
M2R10	1.09 ± 0.08	89.41 ± 2.03	89	52.84	1.37
M2R20	1.55 ± 0.14	176.70 ± 2.55	88	55.73	1.76

^1^mean (*n* = 3) ± standard deviation; ^2^mean (*n* = 3).

**Table 4:** Release data obtained by fitting the dissolution profiles of pure resveratrol and PHBV/PCL microparticles to the biexponential equation.

Material	Biexponential model			
	MSC	*R*	*α* (min^−1^)	*β* (min^−1^)
Resveratrol	5.45	0.9988	0.9902	0.8262
M1R5	5.30	0.9984	0.0357	0.0054
M1R10	5.23	0.9982	0.0082	0.0082
M1R20	4.26	0.9953	0.0360	0.0042
M2R5	2.18	0.9616	0.0693	0.0014
M2R10	4.32	0.9956	0.0465	0.0044
M2R20	4.18	0.9949	0.0363	0.0066

## References

[1] Karacay Ö, Sepici-Dincel A, Karcaaltincaba D (2010). A quantitative evaluation of total antioxidant status and oxidative stress markers in preeclampsia and gestational diabetic patients in 24–36 weeks of gestation. *Diabetes Research and Clinical Practice*.

[2] Pandey KB, Rizvi SI (2010). Resveratrol may protect plasma proteins from oxidation under conditions of oxidative stress in vitro. *Journal of the Brazilian Chemical Society*.

[3] Premkumar K, Bowlus CL (2003). Ascorbic acid reduces the frequency of iron induced micronuclei in bone marrow cells of mice. *Mutation Research—Genetic Toxicology and Environmental Mutagenesis*.

[4] Barreiros ALBS, David JM, David JP (2006). Estresse oxidativo: relação entre geração de espécies reativas e defesa do organismo. *Quimica Nova*.

[5] Shi G, Rao L, Yu H, Xiang H, Yang H, Ji R (2008). Stabilization and encapsulation of photosensitive resveratrol within yeast cell. *International Journal of Pharmaceutics*.

[6] Norata GD, Marchesi P, Passamonti S, Pirillo A, Violi F, Catapano AL (2007). Anti-inflammatory and anti-atherogenic effects of cathechin, caffeic acid and *trans*-resveratrol in apolipoprotein E deficient mice. *Atherosclerosis*.

[7] Shenouda NS, Zhou C, Browning JD (2004). Phytoestrogens in common herbs regulate prostate cancer cell growth in vitro. *Nutrition and Cancer*.

[8] Jarolim S, Millen J, Heeren G, Laun P, Goldfarb DS, Breitenbach M (2004). A novel assay for replicative lifespan in *Saccharomyces cerevisiae*. *FEMS Yeast Research*.

[9] Pacifici GM (2004). Inhibition of human liver and duodenum sulfotransferases by drugs and dietary chemicals: a review of the literature. *International Journal of Clinical Pharmacology and Therapeutics*.

[10] King RE, Bomser JA, Min DB (2006). Bioactivity of resveratrol. *Comprehensive Reviews in Food Science and Food Safety*.

[11] Das S, Ng KY (2010). Resveratrol-loaded calcium-pectinate beads: effects of formulation parameters on drug release and bead characteristics. *Journal of Pharmaceutical Sciences*.

[12] Lucas-Abellán C, Fortea I, López-Nicolás JM, Núñez-Delicado E (2007). Cyclodextrins as resveratrol carrier system. *Food Chemistry*.

[13] Baur JA, Sinclair DA (2006). Therapeutic potential of resveratrol: the *in vivo* evidence. *Nature Reviews Drug Discovery*.

[14] Jang M, Cai L, Udeani GO (1997). Cancer chemopreventive activity of resveratrol, a natural product derived from grapes. *Science*.

[15] Nemen D, Lemos-Senna E (2011). Preparação e caracterização de suspensões coloidais de nanocarreadores lipídicos contendo resveratrol destinados á administração cutânea. *Química Nova*.

[16] Kim BK, Lee JS, Oh JK, Park DJ (2009). Preparation of resveratrol-loaded poly(*ε*-caprolactone) nanoparticles by oil-in-water emulsion solvent evaporation method. *Food Science and Biotechnology*.

[17] Piñeiro Z, Palma M, Barroso CG (2006). Determination of *trans*-resveratrol in grapes by pressurised liquid extraction and fast high-performance liquid chromatography. *Journal of Chromatography A*.

[18] Trela BC, Waterhouse AL (1996). Resveratrol: isomeric molar absorptivities and stability. *Journal of Agricultural and Food Chemistry*.

[19] Lu Z, Cheng B, Hu Y, Zhang Y, Zou G (2009). Complexation of resveratrol with cyclodextrins: solubility and antioxidant activity. *Food Chemistry*.

[20] Frozza RL, Bernardi A, Paese K (2010). Characterization of *trans*-resveratrol-loaded lipid-core nanocapsules and tissue distribution studies in rats. *Journal of Biomedical Nanotechnology*.

[21] Oganesyan EA, Miroshnichenko II, Vikhrieva NS, Lyashenko AA, Leshkov SY (2010). Use of nanoparticles to increase the systemic bioavailability of *trans*-resveratrol. *Pharmaceutical Chemistry Journal*.

[22] Peng H, Xiong H, Li J (2010). Vanillin cross-linked chitosan microspheres for controlled release of resveratrol. *Food Chemistry*.

[23] Riekes MK, Barboza FM, Vecchia DD (2011). Evaluation of oral carvedilol microparticles prepared by simple emulsion technique using poly(3-hydroxybutyrate-*co*-3-hydroxyvalerate) and polycaprolactone as polymers. *Materials Science and Engineering C*.

[24] ICH-Harmonised Tripartity Guideline (2005). *Validation of Analytical Procedures: Methodology*.

[25] Khan KA (1975). The concept of dissolution efficiency. *Journal of Pharmacy and Pharmacology*.

[26] Beck RCR, Pohlmann AR, Benvenutti EV, Costa TD, Guterres SS (2005). Nanostructure-coated diclofenac-loaded microparticles: preparation, morphological characterization, *in vitro* release and *in vivo* gastrointestinal tolerance. *Journal of the Brazilian Chemical Society*.

[27] Siepmann J, Peppas NA (2001). Modeling of drug release from delivery systems based on hydroxypropyl methylcellulose (HPMC). *Advanced Drug Delivery Reviews*.

[28] Dypbukt JM, Bishop C, Brooks WM, Thong B, Eriksson H, Kettle AJ (2005). A sensitive and selective assay for chloramine production by myeloperoxidase. *Free Radical Biology and Medicine*.

[29] Re R, Pellegrini N, Proteggente A, Pannala A, Yang M, Rice-Evans C (1999). Antioxidant activity applying an improved ABTS radical cation decolorization assay. *Free Radical Biology and Medicine*.

[30] Li S, Irvin GC, Simmons B (2000). Structured materials syntheses in a self-assembled surfactant mesophase. *Colloids and Surfaces A*.

[31] Hapner CD, Deuster P, Chen Y (2010). Inhibition of oxidative hemolysis by quercetin, but not other antioxidants. *Chemico-Biological Interactions*.

[32] Banerjee A, Kunwar A, Mishra B, Priyadarsini KI (2008). Concentration dependent antioxidant/pro-oxidant activity of curcumin. Studies from AAPH induced hemolysis of RBCs. *Chemico-Biological Interactions*.

[33] Bosquillon C, Rouxhet PG, Ahimou F (2004). Aerosolization properties, surface composition and physical state of spray-dried protein powders. *Journal of Controlled Release*.

[34] Hung CF, Fang CL, Liao MH, Fang JY (2007). The effect of oil components on the physicochemical properties and drug delivery of emulsions: tocol emulsion versus lipid emulsion. *International Journal of Pharmaceutics*.

[35] Maghsoodi M (2009). Physicomechanical properties of naproxen-loaded microparticles prepared from eudragit L100. *American Association of Pharmaceutical Scientists*.

[36] Farago PV, Raffin RP, Pohlmann AR, Guterres SS, Zawadzki SF (2008). Physicochemical characterization of a hydrophilic model drug-loaded PHBV microparticles obtained by the double emulsion/solvent evaporation technique. *Journal of the Brazilian Chemical Society*.

[37] Duarte MAT, Hugen RG, Martins ES, Pezzin APT, Pezzin SH (2006). Thermal and mechanical behavior of injection molded poly(3-hydroxybutyrate/poly(*ε*-caprolactone) blends. *Materials Research*.

[38] Stulzer HK, Silva MAS, Fernandes D, Assreuy J (2008). Development of controlled release captopril granules coated with ethylcellulose and methylcellulose by fluid bed dryer. *Drug Delivery*.

[39] Desai MP, Labhasetwar V, Amidon GL, Levy RJ (1996). Gastrointestinal uptake of biodegradable microparticles: effect of particle size. *Pharmaceutical Research*.

[40] Chew NYK, Chan HK (2002). Effect of powder polydispersity on aerosol generation. *Journal of Pharmacy and Pharmaceutical Sciences*.

[41] Poletto FS, Jager E, Ré MI, Guterres SS, Pohlmann AR (2007). Rate-modulating PHBHV/PCL microparticles containing weak acid model drugs. *International Journal of Pharmaceutics*.

[42] Ansari KA, Vavia PR, Trotta F, Cavalli R (2011). Cyclodextrin-based nanosponges for delivery of resveratrol: *in vitro* characterisation, stability, cytotoxicity and permeation study. *American Association of Pharmaceutical Scientists*.

[43] Stulzer HK, Tagliari MP, Parize AL, Silva MAS, Laranjeira MCM (2009). Evaluation of cross-linked chitosan microparticles containing acyclovir obtained by spray-drying. *Materials Science and Engineering C*.

[44] Kim S, Ng WK, Dong Y, Das S, Tan RBH (2012). Preparation and physicochemical characterization of *trans*-resveratrol nanoparticles by temperature-controlled antisolvent precipitation. *Journal of Food Engineering*.

[45] Sun X, Shao Y, Yan W (2008). Measurement and correlation of solubilities of *trans*-resveratrol in ethanol+water and acetone+water mixed solvents at different temperatures. *Journal of Chemical and Engineering Data*.

[46] Fortunato KA, Doile MM, Schmücker IC, Schucko SK, Silva MAS, Rodrigues PO (2007). Influência da complexação com *β*-ciclodextrina sobre a liberação do acetato de dexametasona a partir de matrizes hidrofílicas de hidroxipropilmetilcelulose (HPMC) e polioxetileno (PEO). *Latin American Journal of Pharmacy*.

[47] Raffin RP, Colomé LM, Pohlmann AR, Guterres SS (2006). Preparation, characterization, and *in vivo* anti-ulcer evaluation of pantoprazole-loaded microparticles. *European Journal of Pharmaceutics and Biopharmaceutics*.

[48] Cótica LF, Freitas VF, Dias GS (2012). Simple and facile approach to synthesize magnetite nanoparticles and assessment of their effects on blood cells. *Journal of Magnetism and Magnetic Materials*.

[49] Jung HJ, Seu YB, Lee DG (2007). Candicidal action of resveratrol isolated from grapes on human pathogenic yeast *C. albicans*. *Journal of Microbiology and Biotechnology*.

